# Supplementary material for: Development of COVID-19 Vaccine Candidates Using Attenuated Recombinant Vesicular Stomatitis Virus Vectors with M Protein Mutations
Source: Viruses. 2025 Jul 30;17(8):1062. doi: 10.3390/v17081062 (PMC12390721; doi:10.3390/v17081062)
Supplement: Supplementary file 1 [file viruses-17-01062-s001.zip › viruses-3688397-supplementary.pdf]

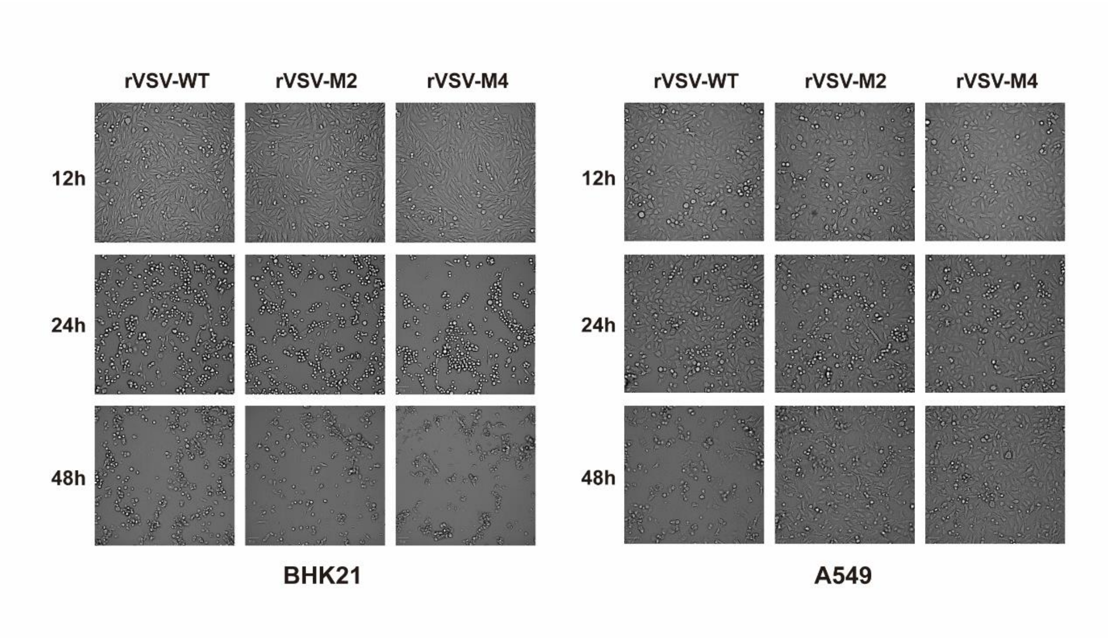

Figure S1. Cytopathic effects following viral infection of BHK21 and A549. BHK21 and A549 cells were infected with rVSV-WT, rVSV-M2 or rVSV-M4 at a MOI of 0.0001. The cytopathic effects were observed at 12, 24, and 48 hours post-infection.

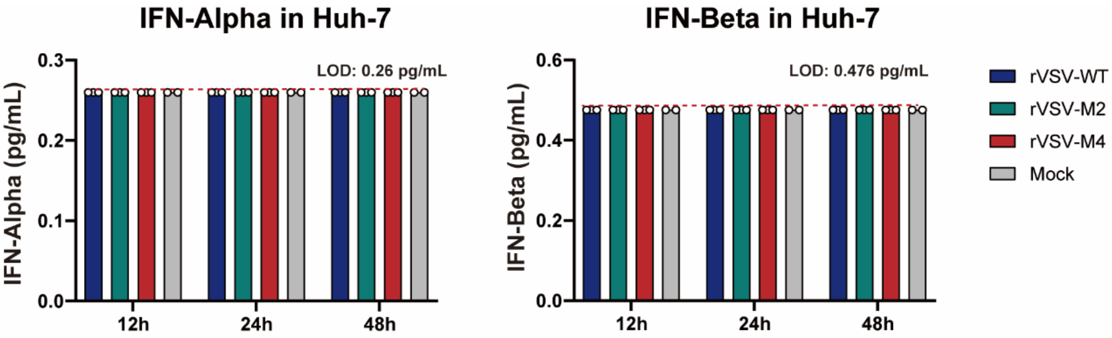

Figure S2. Interferon response detection in Huh-7 cells. Huh-7 cells were infected with rVSV-WT, rVSV-M2 or rVSV-M4 at a MOI of 0.0001. The supernatants were harvested at various time points. INF-α and INF-β levels in all groups were below the detection limit.

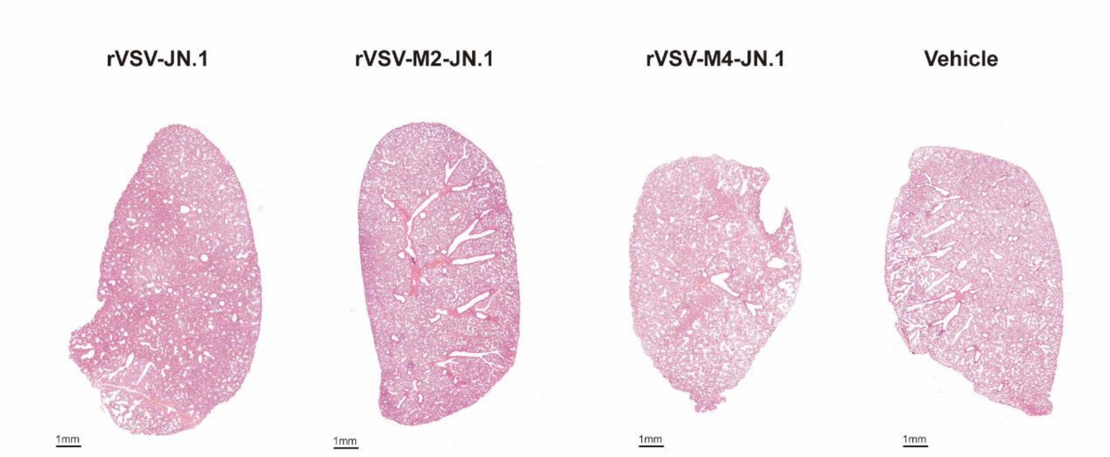

Figure S3. Pathological changes in hamster lung tissue. The overall lung morphology was examined under low magnification.
